# Safety and Tolerability of Essential Oil from *Cinnamomum zeylanicum* Blume Leaves with Action on Oral Candidosis and Its Effect on the Physical Properties of the Acrylic Resin

**DOI:** 10.1155/2014/325670

**Published:** 2014-12-09

**Authors:** Julyana de Araújo Oliveira, Ingrid Carla Guedes da Silva, Leonardo Antunes Trindade, Edeltrudes Oliveira Lima, Hugo Lemes Carlo, Alessandro Leite Cavalcanti, Ricardo Dias de Castro

**Affiliations:** ^1^Post-Graduate Program in Dentistry, Federal University of Paraíba, Campus I, João Pessoa, PB, Brazil; ^2^Post-Graduate Program in Dentistry, State University of Paraíba, Campina Grande, PB, Brazil

## Abstract

The anti-*Candida* activity of essential oil from *Cinnamomum zeylanicum* Blume, as well as its effect on the roughness and hardness of the acrylic resin used in dental prostheses, was assessed. The safety and tolerability of the test product were assessed through a phase I clinical trial involving users of removable dentures. Minimum inhibitory concentration (MIC) and minimum fungicidal concentrations (MFC) were determined against twelve *Candida* strains. Acrylic resin specimens were exposed to artificial saliva (GI), *C. zeylanicum* (GII), and nystatin (GIII) for 15 days. Data were submitted to ANOVA and Tukey posttest (*α* = 5%). For the phase I clinical trial, 15 healthy patients used solution of *C. zeylanicum* at MIC (15 days, 3 times a day) and were submitted to clinical and mycological examinations. *C. zeylanicum* showed anti-*Candida* activity, with MIC = 625.0 *µ*g/mL being equivalent to MFC. Nystatin caused greater increase in roughness and decreased the hardness of the material (*P* < 0.0001), with no significant differences between GI and GII. As regards the clinical trial, no adverse clinical signs were observed after intervention. The substance tested had a satisfactory level of safety and tolerability, supporting new advances involving the clinical use of essential oil from *C. zeylanicum.*

## 1. Introduction

Denture stomatitis is an inflammatory reaction in the oral tissues in contact with dentures and is often reported as being opportunistic infection, mainly caused by fungi of the genus* Candida*. In addition, they are more prevalent in patients using prostheses, immunosuppressed individuals, and chronic users of antimicrobial agents [1–3]. Clinically, it can be characterized as an erythematous area of well-defined contours, present in regions of contact between the dental prosthesis and oral cavity mucosa, causing changes in the texture of surfaces on which it develops [4, 5].

It is known that infection is related to contamination of the inner surface of the denture, since it is considered a reservoir of microorganisms due to irregularities and pores that develop on the surface of acrylic resin denture base materials [6–8]. In this context, the treatment of denture stomatitis lesions involves providing patients with guidance on denture and oral cavity cleaning, removal of potential irritant factors (poorly fitted and unsatisfactory dental prostheses), and use of antifungal agents and the manufacture of a new denture [9, 10].

However, it is known that this type of candidosis is not easy to treat, since relapses often occur after discontinuation of drug therapy, even if the traumatic factor has been eliminated by replacement with a new denture. Thus, treatment of this pathology is a challenge to clinical practice, given the intense frequency of drug administration, small number of drugs available, and increasing resistance expressed by microorganisms [11, 12].

Due to these undesirable factors and the relative toxicity present in conventional antimycotics, there is growing interest in the study of plants with therapeutic properties and antimicrobial activity, not only because they offer an alternative therapeutic approach, but also due to the perspective of isolating substances with significant efficacy against pathogenic fungi [13, 14].

One of the plant species recognized for its biological properties is* Cinnamomum zeylanicum *(cinnamon) [15]. Experimental* in vitro* and* in vivo* studies in animals and humans, conducted in different regions of the world, have shown numerous beneficial effects of* C. zeylanicum *on health [16]. Its analgesic, antiseptic, antispasmodic, astringent, insecticidal, and antimicrobial properties are among the main indications for its use [17–20]. These studies are linked to the idea of the low toxicity of extracts obtained from this plant, considering that it is used as food by human populations and that previous investigations in animals indicated no significant toxic effects, with wide therapeutic potential [16, 21], with reported hepatoprotective [22] and antioxidant effects [23, 24].

Among the properties required of materials used in the manufacture of dental prostheses, those related to surface roughness, surface tension, electrostatic interactions, and hardness are of clinical importance. According to the authors, surface roughness causes adhesion and retention of* Candida albicans*, which is of particular importance for the induction of stomatitis [25]. In this sense, it is relevant to determine the effects of antifungal agents on the acrylic resin used in the manufacture of dental prostheses, since the treatment protocol of oral candidiasis requires the product to be applied on the affected mucosa; thus it reaches the denture base.

Although there are reports on the antimicrobial activity of essential oils obtained from* C. zeylanicum* [26], clinical trials involving humans are scarce. Preclinical information can, however, be obtained from* in vitro* studies or with animal models; nevertheless, the first clinical tolerability data should also be obtained in humans [16]. The proposal of using* C. zeylanicum* in the treatment of oral candidiasis provides external use of the product (topical), which must be applied to affected oral mucosa without ingesting it, thus minimizing systemic effects. However, signs and symptoms resulting from the use of the product must be assessed by means of studies that include a phase I clinical study design. This generally includes healthy people in order to identify possible adverse effects, whereas phase II clinical studies, in turn, include unhealthy volunteers randomly distributed between test group and control group in order to obtain comparative efficacy data from a test substance and a standard substance, which has been well established in the literature [27].

Given the scarcity of studies addressing the antimicrobial properties of medicinal plants used in dentistry, in the present study, the action of essential oil from* C. zeylanicum* leaves was evaluated by assessing its antifungal activity and the possible changes caused by the test substance on the physical properties of heat-polymerized acrylic resin, which is the basic material of dentures. In a subsequent step, the study also sought to clinically investigate the safety and tolerability of the test product by means of a phase I clinical trial.

## 2. Material and Methods

### 2.1. Ethical Considerations and Trial Registration

The study was approved by the Research Ethics Committee of the Federal University of Paraíba and registered in the Brazilian Registry of Clinical Trials (http://www.ensaiosclinicos.gov.br/).

### 2.2. Study Design

This research was divided into three stages: an* in vitro *microbiological analysis of the antifungal activity of the test substance; effect of the test product on the physical properties of the acrylic resin; and phase I clinical trial investigating the safety and tolerability of the test product.

### 2.3. *In Vitro* Assays

Essential oil from* C. zeylanicum* leaves (cinnamon) (Ferquima Ind. e Com., Vargem Grande Paulista, São Paulo, Brazil) with characteristic appearance, color, odor, density, and refractive indexes (20°C) of 1.043 and 1.533, respectively, was used.

For chemical characterization of the test substance, chemical analysis by chromatography was performed using a gas chromatograph coupled to a mass spectrometer (Shimadzu GC-MS-QP5050A) and capillary column (J & W SCIENTIFIC) with stationary phase of 5% phenyl and 95% dimethylpolysiloxane, measuring 30 m long, 0.25 mm internal diameter, and 0.25 *µ*m of film thickness. The initial temperature programming ranged from 60°C to 240°C (3°C/min), while the programmed running time was 60 minutes, and the oven temperature was 250°C. Helium was used as carrier gas (mobile phase) at a flow rate of 1.0 mL/min with a 1 : 20 split ratio and injection volume of 1 *μ*L. The compounds were identified by comparing their mass spectra with those existing in the database of the equipment (MIST Library, 2008). The essential oil sample was injected at a concentration of 2 ppm and hexane was used as solvent. The chromatogram and mass spectra were analyzed with the aid of a device library and the integration parameters used were width: 3 and slope: 2000.

A standard drug, antifungal nystatin (Sigma-Aldrich Brazil Ltda., São Paulo, Brazil), in the form of powder was used as positive control. The solutions were prepared at the time of performing the tests.

*Step I: In Vitro Verification of the Antifungal Activity of Essential Oil from C. zeylanicum and Nystatin.* Consider the following.

*(I) Determination of the Minimum Inhibitory Concentration (MIC).* MIC was determined using the microdilution technique [28] in 96-well plates that received 100 *µ*L of Sabouraud Dextrose Broth (Difco Laboratories, Detroit, Mich., USA). Then, 100 *µ*L of the test substance at an initial concentration of 5.000 *μ*g/mL was distributed as follows: serial dilution was performed by collecting an aliquot of 100 *µ*L from a cavity containing a more concentrated substance to the following cavity. Sequentially, 10 *μ*L aliquots of the inoculum corresponding to 12 strains tested were dispensed in the holes of each column, 8 strains of* Candida albicans*, and 4 of* Candida tropicalis* (*C. albicans* ATCC 76485;* C. albicans* ATCC 76645;* C. albicans *LMP20;* C. albicans *LM111;* C. albicans *LM62;* C. albicans *LM108;* C. albicans *LM122;* C. albicans *LM86;* C. tropicalis *ATCC13803;* C. tropicalis *LM45;* C. tropicalis *LM14;* C. tropicalis *LM20).

In addition, control of the viability of yeast tested (negative control) and positive control against the antifungal action of nystatin were performed. The assays were conducted in duplicate and incubated at 35°C for 48 hours, at approximately 80% of humidity. The reading of the dilution microplates for MIC determination of test strains was performed by the visual method. To confirm the presence or absence of viable microorganisms in noninhibitory concentrations, a volume of 10 *μ*L of TTC dye (2,3,5-triphenyl tetrazolium chloride) was used. This dye is able to reflect the activity of dehydrogenase enzymes involved in the cellular respiration process, which makes it possible to distinguish live samples that are stained in red from dead ones, which maintained the original color [29].

*(II) Determination of the Minimum Fungicidal Concentration (MFC).* After MIC determination, the concentration corresponding to the inhibitory concentration (MIC) and the two concentrations immediately more concentrated (MICx2 and MICx4) as well as the positive controls were subcultured on Sabouraud dextrose agar (SDA) plates (Difco Laboratories, Detroit plates, Mich., USA) containing 10 *μ*L of inoculum. After 24 h of incubation at 30°C, the readings of MFCs were performed based on the growth of controls, and the MFC was considered the lowest drug concentration that prevented visible growth of the subculture [28].

*Step II: Roughness and Microhardness Test.* Consider the following.

*(I) Fabrication of Specimens.* Thirty heat-polymerized acrylic resin specimens were prepared (Destac Dent, Pirassununga, São Paulo, SP, Brazil) using a silicone matrix of dense condensation (Flexitime, São Paulo, SP, Brazil), measuring 2 mm in thickness and 4 mm in diameter.

The specimens were placed in distilled water at 37°C for one hour, exposed to dry heat in an oven for 24 hours to remove residual monomer, and polished with carborundum sandpaper (numbers 220, 330, 600, and 1200), felt discs and pumice stone/distilled water paste. Subsequently, they were washed in running water for 30 seconds [30, 31].

*(II) Determination of Changes in Surface Roughness and Microhardness.* The specimens were divided into three groups (*n* = 10): GI: artificial saliva (negative control), obtained from the pharmacy (Table 2), GII: mouthwash with addition of* C. zeylanicum* (composed of distilled water, Tween 80, and essential oil from* C. zeylanicum* at MIC), and GIII: nystatin 100,000 UI/mL.

Group I was kept in artificial saliva throughout the test, while groups II and III were immersed for 1 minute, 3 times a day, in their corresponding solutions (mouthwash and nystatin, resp.) and then in artificial saliva for 15 days, during which the system remained incubated in a biological oven in the dark at 37°C. Initial and final measurements were performed (24 hours before and 24 hours after the start and end of the test, resp.) in order to evaluate possible changes in the mechanical properties of the heat-polymerized acrylic resin.

For the roughness trial, the samples received markings on one of the surfaces, corresponding to the middle region, 1 mm to the right and 1 mm to the left, and were evaluated (SJ-201 Minutayo, Kawasaki, Japan), according to the ISO 1997 standard (speed 0.5 mm/s, *λc* 0.8, and distance 1) by which initial and final measurements were performed. For each sample, the arithmetic mean of the three values obtained was calculated, which was considered the surface roughness in micrometers (Ra) [32, 33].

For the hardness test, the same samples were used; however, the opposite surface to that intended for the roughness test was measured. Microhardness values were measured before and after the test. A microhardness tester (Shimadzu, Kyoto, Japan) containing a Vickers type diamond tip indenter was used in this step, applying 25 g of force for 30 seconds. For each specimen, five indentations were performed, and the unit of results was obtained in Vickers hardness number (VHN) after calculating the mean value obtained after five measurements of each sample [34, 35].

### 2.4. Clinical Stage: Phase I Clinical Trial

#### 2.4.1. Inclusion and Exclusion Criteria

The study included healthy individuals aged 40–60 years, users of removable full or partial maxillary dentures. Patients who had been under treatment with antimicrobial agents for at least 6 months before the study and those with a history of sensitivity to cinnamon were excluded.

#### 2.4.2. Sample

The sample consisted of 15 patients treated in public service health units. Anamnesis and clinical examinations were performed by two calibrated investigators. *K* values obtained in the calibration were 0.75 and 1 for inter- and intraexaminer, respectively, representing good and excellent agreement [36].

#### 2.4.3. Clinical and Mycological Diagnosis of Denture Stomatitis

Anamnesis and preliminary clinical examinations were performed to check the absence or presence of signs and symptoms of denture stomatitis, such as change in mucosa texture, presence of whitish areas susceptible to removal by scraping, erythematous mucosa underlying the dental prosthesis, pain, burning, itching, and/or halitosis. Furthermore, a mycological laboratory test was performed by collecting material from the oral mucosa and subsequent cultivation in culture medium. Biological samples (secretion and scales from the palatal mucosa) were collected with a sterile swab, allocated into identified test tubes containing saline (0.85%) solution, and sent to the Laboratory of Clinical Mycology, CCS/UFPB, for processing and diagnosis. Sequentially, the samples were grown in culture medium (Chromagar* Candida*, Difco Laboratories, Detroit, USA) through a calibrated loop of 1 *μ*L and incubated at room temperature for 48 hours, when the reading was taken.

After obtaining the laboratory results of mycological examination, volunteers with negative diagnosis (clinical and mycological) of candidiasis were recruited and submitted to antifungal therapy, which consisted of administration of a mouthwash based on* C. zeylanicum* (cinnamon) at MIC, with the following composition: distilled water, essential oil of* C. zeylanicum,* and Tween 80.

Personal and family history, allergies and hypersensitivities, harmful habits, hygiene habits, and use of drugs, as well as diagnostic information and possible adverse effects, were recorded on a medical record chart before and after the administration of the product. The photographic record of the palate of participants as well as of their dental prostheses was also performed in two time intervals, with the aid of orthodontic mirror and SONY camera (16.1 mega pixels DSC, W570D) in order to serve as an auxiliary tool for qualitative analysis of tolerability and adverse reactions, including the following signs and symptoms: burning, taste alterations, itching, peeling, erythema, and color change of teeth and/or dentures.

#### 2.4.4. Intervention Protocol

Subjects were instructed to use mouthwash 3 times a day for a period of 15 days after cleaning the oral cavity, performing mouth washes with 10 mL for about 60 seconds, without ingesting the product. They were also informed that, after the use of mouthwash, they should not eat any food and should drink no water for 30 minutes.

The participants received a container containing 500 mL of test mouthwash, an injector (10 mL), a stiff bristle tooth brush (Colgate Classic), and toothpaste (Colgate Triple Action) to use for cleaning the dental prostheses. Subsequently, they were also provided with written instructions containing two numbers for contacts with the head researcher in cases of doubt or communication of adverse reaction. In case of the occurrence of unwanted events, the volunteers were instructed to promptly discontinue the intervention protocol and communicate with the head researcher so that undesirable clinical signs and symptoms could be recorded.

During all the days of intervention, two researchers maintained at least one daily verbal contact with participants by phone calls, reminding them to comply with the schedule and of the correct way to use the mouthwash. Twenty-four hours before and after use of the test product, anamnesis and clinical examination were performed accompanied by laboratory mycological examination.

### 2.5. Statistical Analysis

The database was compiled in the GraphPrisma statistical software program version 6.0. A descriptive statistical analysis of laboratorial clinical and microbiological data was performed. For roughness and hardness trials, ANOVA associated with posttest Tukey test was used, adopting a significance level of 5%.

## 3. Results

**Figure 1:**
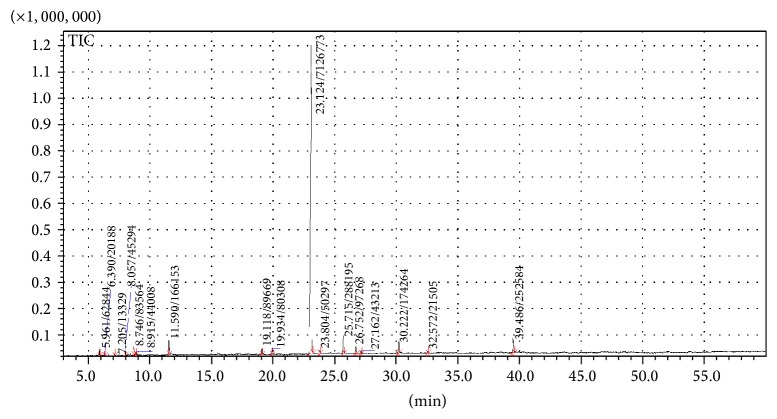
Chromatogram of* Cinnamomum zeylanicum* essential oil.

The chromatographic analysis of the essential oil from* C. zeylanicum* leaves allowed the identification of seventeen chromatographic peaks (Table 1 and Figure 1). With regard to the major component, eugenol obtained a relative concentration of 82.30%, with peak at time of 23.3 minutes.

The results of the minimum inhibitory concentration (MIC) and minimum fungicidal concentration (MFC) of the essential oil from* C. zeylanicum* and nystatin are shown in Table 2.

It was found that all test strains showed sensitivity to the action of the essential oil, and the concentration of 625.0 *µ*g/mL was able to inhibit all samples. Three types of yeast (*C. albicans* ATCC 76485,* C. albicans *ATCC 76645, and* C. albicans *LM 62) were inhibited at concentration of 312.5 *µ*g/mL, showing the most sensitive behavior to the action of* C. zeylanicum*.

Among the test strains, eleven showed no microbial growth at a concentration equivalent to that found during MIC determination, equivalent to MFC in 91.66% of the* Candida* sample. The only yeast corresponding to* C. albicans* ATCC 76485 showed MFC equal to MICx2, however, with the value of 625.0 *µ*g/mL remaining as fungicide concentration. It was observed that the concentration of 625 *µ*g/mL was able to prevent microbial growth of all yeast and was also considered the MFC.

Used as a positive control, nystatin also had MIC and MFC values determined (Table 2). Among the test strains, nine demonstrated growth inhibition at concentration of 8 *µ*g/mL. Two strains (*C. tropicalis* LM 14 and* C. tropicalis* LM 20) were less sensitive to the action of nystatin, with MIC of 16 *μ*g/mL, while the sample corresponding to* C. albicans* ATCC 76485 required a higher concentration of the control antifungal agent in order to inhibit its growth (MIC = 32 *μ*g/mL).

During the performance of tests to determine the MFC of nystatin, 8 strains equivalent to 66.6% of the sample showed MFC of 8 *µ*g/mL equivalent to MIC. The same equivalence was observed for yeast* C. tropicalis* LM 20, with MIC and MFC values being equal to 16 *μ*g/mL.* C. tropicalis *LM 45 and* C. tropicalis* and LM 14 showed MFC values of 16 *μ*g/mL and 64 *μ*g/mL.* C. albicans* ATCC 76485 showed a higher fungicidal concentration (128 *μ*g/mL), which is 4 times the value of the MIC.

**Figure 2:**
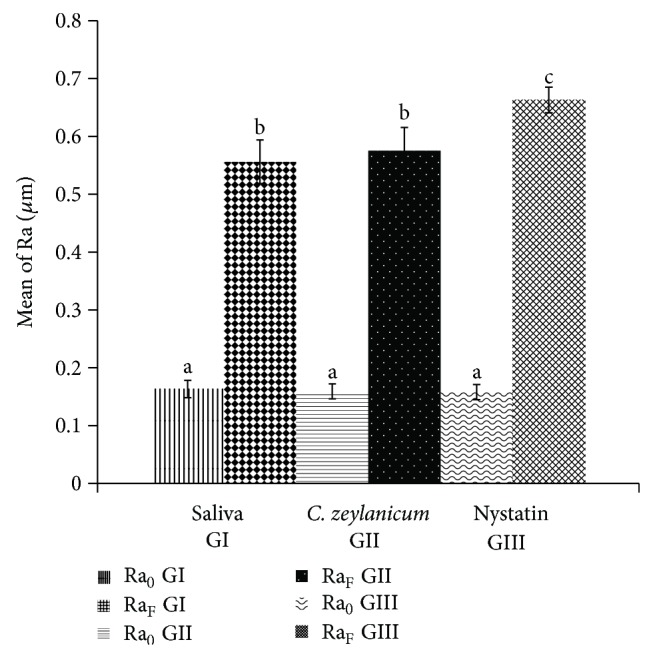
Changes in surface roughness (Ra) before and after treatments. RA_0_ is initial roughness and Ra_F_ is final roughness; different letters indicate statistically significant differences, while equal letters indicate absence of statistical significance.

Figure 2 shows the results of mean roughness for GI, GII, and GIII before and after intervention, and no differences were identified between initial measurements of specimens in GI, GII, and GIII (*P* > 0.05). However, significant changes in roughness were observed in the three groups, when their measurements before and after treatment were compared.

In addition to the statistical significance with respect to surface roughness (Ra) between pre- and posttreatment values in the same group, Figure 1 also showed significant differences in final Ra measures when comparing pairs “GI: saliva/GIII: nystatin” and “GII:* C. zeylanicum*/GIII: nystatin.” It was observed that the negative control (GI: saliva) showed a smaller increase of surface roughness (0.39 *µ*m) in comparison with the positive control (GIII: nystatin), in which larger changes occurred (0.50 *µ*m) in this property (*P* < 0.0001).

Although the trial of the mouthwash based on* C. zeylanicum* also showed interference in the surface roughness of samples before and after treatment (0.40 *µ*m), these values were lower than those shown for the standard antifungal agent, consistent with nystatin intervention (*P* < 0.0001), similar to GI. There was no statistical difference between final roughness measurements (Ra_F_) and roughness variation (ΔRa) of GII (*C. zeylanicum*) and GI (saliva).

**Figure 3:**
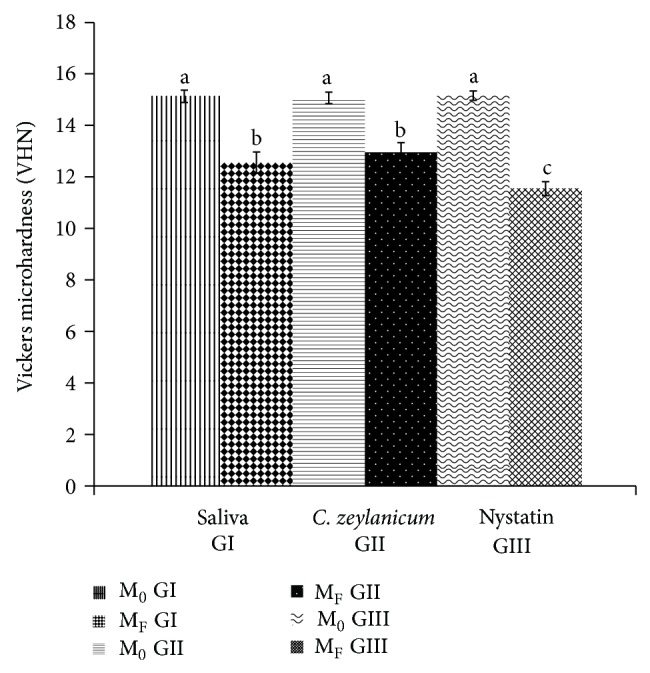
Changes in Vickers microhardness before and after treatments. M_0_ is initial microhardness and M_F_ is final microhardness; different letters indicate statistically significant differences, while equal letters indicate absence of statistical significance.

Figure 3 shows the results of the mean microhardness values of five indentations performed for each specimen in GI, GII, and GIII, before and after intervention. No significant differences between baseline values were observed between groups (*P* > 0.05), demonstrating a standardization of the initial samples for the three groups and the minimization of methodological biases. However, there were changes in the three groups (*P* < 0.0001) when initial and final mean values before and after treatment were compared.

It was observed that the groups consisting of the test substance (GII) and saliva (GI) caused a smaller decrease in microhardness (−2.12 VHN and −2.57 VHN, resp.), with no statistical difference between them. It was found, however, that the group consisting of nystatin was capable of producing greater interference in the measured property (−3.62 VHN), which is significant in comparison with the other groups (*P* < 0.0001). These results were positive in comparison with the test substance, since the influence on the decrease in microhardness of the test substance was less than that of nystatin.

### 3.1. Results regarding the Phase I Clinical Trial

#### 3.1.1. Sample Characterization

After conducting the clinical intervention protocol for 15 days, patients were once again submitted to clinical and mycological examination (in which patients were investigated for alteration of palatal mucosa, erythema, edema, milky white plates, and hyperplasia) 24 hours after the end of last mouthrinse. The users were also asked about the presence of any adverse symptoms such as itching, burning, discomfort, loss of taste, and complaints about some modifications in the prosthesis.

After using the test mouthwash, at a concentration of 625 *μ*g/mL (adopted from results obtained in the determination of the minimum inhibitory concentration of* C. zeylanicum* observed in the laboratory phase of this study), none of the participants showed clinical signs of color, shape, or texture change in the area of mucosa that supports the denture. With regard to symptoms, only one of the fifteen patients complained of a slight “burning” in the region of the tongue after using the mouthwash; however, the patient reported that this complaint was of short duration (about 2 minutes after use). On mycological examination, all patients were negative for the growth of fungal colonies on a solid medium.

## 4. Discussion

The genus* Cinnamomum* includes about 250 species, which are found in nature in the form of shrubs and small to medium sized trees. They can be found in tropical forests, growing in well-drained plains and mountains. The genus is found in abundance in Southeastern East Asia, where species* C. zeylanicum, C. loureiroi, C. burmannii,* and* C. cassia* are the most used by the population, and the essential oil obtained from bark and leaves is used as food flavor, in perfumery and also in pharmaceutical preparations [37–39].

The clinical use of natural products with action on oral candidiasis has rarely been reported in literature, and there is a lack of clinical research with essential oil obtained from* C. zeylanicum* leaves for this purpose. The use of essential oil extracted from the bark of this plant for the treatment of this lesion was described by a single trial, characterized as a pilot study, as it included only five individuals [18]. Essential oils from other plant species have not been studied from a clinical perspective for the treatment of oral candidiasis.

With respect to studies involving the pharmaceutical use or the antimicrobial activity of natural products, it should be stressed that these uses must preferably be associated with a chemical analysis of the test substance [40]. This practice provides the identification of key substances, suggesting a possible biological effect on the microorganisms involved in the disease.

Previous studies have indicated that the essential oil from* C. zeylanicum* leaf has significant amounts of eugenol [41, 42], corroborating the chromatographic results shown. The antifungal potential of the essential oil from* C. zeylanicum* bark is associated with high levels of cinnamaldehyde [43] and eugenol [39, 42], although other constituents may also contribute to the antimycotic activity.

Evaluation of the effect of phytochemicals (eugenol, cinnamaldehyde, citral, and geraniol) on* C. albicans* biofilm has shown that their inhibitory activity is dose- and time-dependent and is positively influenced by the presence of eugenol [44], which in turn is able to reduce the metabolic activity of mature biofilms and inhibit the adhesion of* Candida *to synthetic surfaces and human epithelial cells [45].

When microorganisms become resistant to therapies adopted, there is an increased incidence of infections and therefore increasing need for constant search for new effective substances [4]. In an attempt to obtain a new alternative treatment for denture stomatitis, laboratory tests of minimum fungicidal inhibitory concentration were performed in this study against twelve* Candida* strains, eight of them being* C. albicans* and four being* C. tropicalis,* because these are the most prevalent in oral fungal infections. These species have specific virulence factors favorable to the formation of biofilms and production of extracellular enzymes such as phospholipases and proteases, capable of promoting the destruction of tissues in the host [46, 47].

*C. albicans* accounted for the largest number of strains selected in this study, which is justified due to its high virulence in comparison with other species. Some studies have suggested that this high virulence is linked to an increased amount of interference in intracellular signaling pathways, such as MAP kinase signal transduction (Mitogen-Activated Protein). These interferences would generate cellular responses involved in invasive growth, cell wall formation, adaptation to osmotic stress, and reproduction [48].

Other studies have also reported the antifungal activity of the essential oil from* C. zeylanicum*, even considering the different methodologies and concentrations of the test product [41–43, 49]. As regards nystatin used as a control in this study, there is evidence to prove its antifungal activity [50].

The chemical configuration of the leading molecules identified in the essential oil, especially monoterpenos such as eugenol, gives them hydrophobic properties. This allows their deposition on the lipophilic structures of microorganisms such as the plasma membrane, leading to increased permeability with consequent loss of electrolytes essential to the survival of fungal cells [51]. Other mechanisms of action such as inhibition of membrane synthesis, spore germination, and cell respiration may also be present [52]. It was observed that terpene alcohols such as linalool can act as desiccants and solvents that cause protein denaturation [53].

Acrylic resins have been the materials of choice for the manufacture of dentures since the 1930s, and their use is supported by its properties of hardness, biocompatibility, lack of taste and odor, and roughness, among others [54, 55]. In this sense, it is necessary to evaluate the effect of therapeutic substances that may come into contact with this material during clinical use, such as in the treatment of oral candidiasis associated with the use of dental prostheses. This is the first study to assess the effect of essential oils from* C. zeylanicum* on the physical properties of the acrylic resin (surface roughness and hardness) used in the manufacture of dentures.

To evaluate the surface roughness of the acrylic resin used in the preparation of dental prostheses, the specimens used in this research were submitted to a process of mechanical polishing and obtained initial mean values of 0.16 *µ*m for the three groups. These results are satisfactory, considering that the surface roughness value of heat-polymerized resins can range from 0.03 *µ*m to 0.75 *µ*m [56]. There was no statistical difference among samples and groups before treatment, revealing a standardization of the initial surface roughness of samples. For final values above 2 *μ*m, there is a significant increase in the colonization of microorganisms on the surface of the acrylic resin [57].

In the present study, there was a significant increase in surface roughness for all groups, including the negative control group (saliva), for which the specimens remained immersed in artificial saliva for fifteen days. The significant increase in surface roughness in the control group can be attributed to sorption and solubility during the immersion process, which has a direct influence on the mechanical properties of the heat-polymerized acrylic resin [58].

Although all groups underwent significant changes in surface roughness in their final measures, there was no significant difference between final roughness of the saliva and test product groups, which had a lower surface roughness change compared with the reference group (nystatin). These results generate positive perspectives for a possible clinical use of mouthwash based on* C. zeylanicum* in further trials.

In order to draw a tolerability profile of the product used, the property with regard to Vickers microhardness was also evaluated for the three groups. Hardness can be defined as the ability of a substance to resist the wear or penetration. Knowledge of the hardness of materials used in dentistry is very important since it is related to the clinical longevity of materials. The hardness test is a nondestructive, specifically located laboratory test, which provides data on the distribution of properties of materials [59].

In this study, specimens showed no statistical differences in initial mean values among groups for Vickers microhardness, suggesting sample standardization. However, there was significant decrease in this property for the three groups after intervention. Although differences were observed in the final measures among groups, in which the mouthwash based on* C. zeylanicum* caused a lower degree of change in hardness when compared with the reference group nystatin, these results demonstrate that although causing changes in the Vickers hardness of the material, these changes were of a lower degree than those caused by the standard product (nystatin) used in clinical practice, thus encouraging the possibility of further studies that promote the suggestion of the test product at MIC, as a tolerable alternative for therapeutic use.

Previous studies [60, 61] have found a significant reduction in the microhardness of the acrylic resin when using other substances (sodium hypochlorite, 4% chlorhexidine gluconate, 2% glutaraldehyde, deionized water, and distilled water) with the aim of disinfecting this material, possibly suggesting that immersion causes changes in the mechanical properties of the material due to the occurrence of sorption and solubility during cycles.

Toxicological studies and phase I clinical trials involving natural products are considered scarce in dentistry, given their importance, so that the scientific evidence shown by* in vitro* assays is able to propose new therapeutic possibilities [62].

The use of natural products in specific situations, such as in dermatology, can often be risky, because they may cause dermatosis due to plants resulting from mechanisms of irritation or photosensitivity [63]. This suggests the importance of conducting a phase I clinical trial for therapies involving the use of natural products, such as that proposed in the present study, in spite of the widespread and well-known use of cinnamon by populations, in the form of spice and flavoring.

For the phase I clinical trial, a sample of 15 subjects was adopted, which was determined from the design proposed for phase I clinical trials involving a small group of healthy volunteers in order to propose or establish a preliminary evaluation of the tolerability of a test product [27].

The results of phase I clinical trial demonstrated a good perspective for the advancement of phase II clinical trials involving* C. zeylanicum* at MIC of 625 *μ*g/mL, since no patient had clinical changes in the mucosa near the dental prosthesis or in other regions of the oral mucosa. As regards symptoms, only one patient reported burning of mild intensity and short duration, and this result is considered satisfactory, given the absence of clinical signs suggestive of any type of acute changes such as erythema or hyperemia, compatible with such symptoms.

Therefore, in view of the foregoing, above and considering the limitations of laboratory steps shown in this research, the study considered the safety and tolerability of the test substance satisfactory, supporting new trials involving the use of essential oil from* C. zeylanicum* in subsequent clinical stages of scientific evidence.

## 5. Conclusion

The essential oil, obtained from* C. zeylanicum* leaves, has activity against* Candida *species, promotes physical changes (roughness and hardness) in the acrylic resin similar to artificial saliva, and presents safety and tolerability when used by healthy individuals subsidizing the development of phase II clinical trial required for the therapeutic investigation of this product in individuals with candidiasis associated with the use of dentures.

## Figures and Tables

**Table 1:** Analytes identified by GC-MS in the essential oil from *Cinnamomum zeylanicum* Blume leaves.

Peak	Retention time	Component	Concentration (%)
Peak 1	5.961	*α*-pinene	0.73
Peak 2	6.390	Camphene	0.23
Peak 3	7.206	*β*-pinene	0.15
Peak 4	8.057	*α*-phellandrene	0.52
Peak 5	8.747	*ρ*-pymene	0.97
Peak 6	8.915	*β*-phellandrene	0.51
Peak 7	11.590	Linalool	1.92
Peak 8	19.118	(Z)-cinnamaldehyde	1.04
Peak 9	19.934	Safrole	0.93
Peak 10	23.124	Eugenol	82.30
Peak 11	23.804	*α*-copaene	0.58
Peak 12	25.715	(E)-caryophyllene	3.33
Peak 13	26.752	(E)-cinnamyl acetate	1.12
Peak 14	27.162	*α*-humulene	0.50
Peak 15	30.222	Eugenyl acetate	2.01
Peak 16	32.572	Caryophyllene oxide	0.25
Peak 17	39.486	Benzyl benzoate	2.92

Total		—	100.00

**Table 2:** Results of MIC and MFC of essential oil from *C. zeylanicum* leaves and nystatin on *Candida* species.

Strains	*C. zeylanicum *		Nystatin	
	MIC (*μ*g/mL)	MFC (*μ*g/mL)	MIC (*μ*g/mL)	MFC (*μ*g/mL)
*C. albicans* ATCC 76485	312.5	625.0	32.0	128.0
*C. albicans* ATCC 76645	312.5	312.5	8.0	8.0
*C. albicans* LM P20	625.0	625.0	8.0	8.0
*C. albicans* LM 111	625.0	625.0	8.0	8.0
*C. albicans* LM 62	312.5	312.5	8.0	8.0
*C. albicans* LM 108	625.0	625.0	8.0	8.0
*C. albicans* LM 122	625.0	625.0	8.0	8.0
*C. albicans* LM 86	625.0	625.0	8.0	8.0
*C. tropicalis* ATCC 13803	625.0	625.0	8.0	8.0
*C. tropicalis* LM 45	625.0	625.0	8.0	16.0
*C. tropicalis* LM 14	625.0	625.0	16.0	64.0
*C. tropicalis* LM 20	625.0	625.0	16.0	16.0
